# HIV-infected Latin American asylum seekers in Madrid, Spain, 2022: A prospective cohort study from a major gateway in Europe

**DOI:** 10.2807/1560-7917.ES.2024.29.29.2300692

**Published:** 2024-07-18

**Authors:** Pablo Ryan, Samuel Manzano, Neda Deihim-Rahampour, Guillermo Cuevas, Laura Martin-Gonzalez, Alicia Gonzalez-Baeza, Pedro Torres, Jeffrey V Lazarus, Juan Torres-Macho, Jorge Valencia, Matilde Sanchez-Conde

**Affiliations:** 1 Hospital Universitario Infanta Leonor, Madrid, Spain; 2 Universidad Complutense de Madrid (UCM), Madrid, Spain; 3 Centro de Investigación Biomédica en Red en Enfermedades Infecciosas (CIBERINFEC), Instituto de Salud Carlos III, Madrid, Spain; 4 Fundación Estatal, Salud, Infancia y Bienestar Social, F.S.P. (FSCAI), Madrid, Spain; 5 Hospital General Universitario Gregorio Marañón (IiSGM), Madrid, Spain; 6 Universidad Autónoma de Madrid (UAM), Madrid, Spain; 7 Fundación para la Investigación e Innovación Biomédica H.U. Infanta Leonor y H.U. Sureste, Madrid, Spain; 8 Barcelona Institute for Global Health (ISGlobal), Hospital Clínic, University of Barcelona, Barcelona, Spain; 9 CUNY Graduate School of Public Health and Health Policy (CUNY SPH), New York, United States; 10 Hospital Universitario Ramon y Cajal, Madrid, Spain; * These authors contributed equally to this work and share first authorship.; ** These authors contributed equally to this work and share last authorship.

**Keywords:** HIV, migrants, Latin American, access to treatment, healthcare barriers, Spain, America, sexually transmitted infections, HIV infection, infection control, public health policy, travel

## Abstract

**Background:**

Recent migration trends have shown a notable entry of Latin American asylum seekers to Madrid, Spain.

**Aim:**

To characterise the profile of asylum-seeking Latin American migrants who are living with HIV in Spain and to outline the barriers they face in accessing HIV treatment.

**Methods:**

A prospective cohort study was conducted between 2022 and 2023 with a 6-month follow-up period. Latin American asylum seekers living with HIV were recruited mainly from non-governmental organisations and received care at an HIV clinic in a public hospital in Madrid.

**Results:**

We included 631 asylum seekers. The primary countries of origin were Colombia (30%), Venezuela (30%) and Peru (18%). The median age was 32 years (interquartile range (IQR): 28–37), and 553 (88%) were cis men of which 94% were men who have sex with men. Upon their arrival, 49% (n = 309) lacked social support, and 74% (n = 464) faced barriers when attempting to access the healthcare system. Upon entry in Europe, 500 (77%) participants were taking antiretroviral therapy (ART). At their first evaluation at the HIV clinic, only 386 (61%) had continued taking ART and 33% (n = 209) had detectable plasma HIV-1 RNA levels. Six months later, 99% took ART and 98% had achieved an undetectable viral load.

**Conclusions:**

Latin American asylum seekers living with HIV in Madrid, Spain encountered barriers to healthcare and to ART. One-third of these individuals presented detectable HIV viral load when assessed in the HIV clinic, highlighting this as an important public health issue.

**Key public health message**

**What did you want to address in this study and why?**

Benefits of immediate antiretroviral therapy (ART) upon diagnosis of HIV are well-documented, however, evidence from studies indicates barriers to healthcare among migrants in high-income countries. Collection of specific data on Latin American asylum seekers living with HIV in Spain are limited, often due to problems associated with data categorisation.

**What have we learnt from this study?**

Our comprehensive analysis of Latin American HIV-positive migrants seeking international protection in Spain highlights the healthcare challenges they encounter, particularly in accessing ART. Despite Spain's universal healthcare and referral systems, one third of asylum seekers in our study showed detectable HIV viral loads during their initial clinical assessments.

**What are the implications of your findings for public health?**

The collective evidence underscores the need for tailored healthcare strategies for Latin American asylum seekers living with HIV in Spain. Recognising and addressing the challenges this population faces in accessing treatment concerns both individual rights and also has broader implications for the public health goal of controlling the HIV/AIDS epidemic.

## Introduction

The human immunodeficiency virus (HIV) continues to pose a major global health challenge [1], although the landscape of HIV infection has changed dramatically since the advent of highly active antiretroviral therapy (ART) [2]. With ART, the risks of disease progression, hospitalisation and death are diminished, thus enhancing individual quality of life and reducing community costs [3-6]. Additionally, since individuals with undetectable viral load due to ART cannot transmit the virus, transmission is effectively halted [7-9]. The World Health Organization (WHO) recommends initiating and maintaining ART for all people living with HIV infection, regardless of CD4^+^ T lymphocyte count [10]. Such an approach requires the implementation of HIV screening programmes and reliable and sustained efforts to ensure immediate and universal access to ART. To this end, the Joint United Nations Programme on HIV/AIDS (UNAIDS) established the 95–95–95 target as a strategy to end the acquired immune deficiency syndrome (AIDS) epidemic by 2030 [11].

Asylum seekers are individuals who have left their country and are seeking protection from persecution or serious harm in another country but whose claim for asylum has not yet been decided, as outlined in the 1951 Refugee Convention [12]. Ensuring that these individuals have access to HIV screening and timely treatment is crucial for both their health and the broader public health goal of controlling the HIV/AIDS epidemic. In 2022, Spain was Europe's primary destination for Latin American asylum seekers, receiving 45,730 applications from Venezuela (90.1% of all Venezuelan applicants who arrived in Europe), 35,910 (83.5%) from Colombia and 8,915 (69.4%) from Peru [13,14]. Evidence suggests that migrants living with HIV that arrive to high-income countries seeking international protection encounter notable challenges in accessing treatment [15-17]. Barriers hindering access to diagnosis, treatments or preventive measures persist despite robust scientific evidence advocating for immediate initiation of ART upon a diagnosis of HIV infection.

Comprehensive data on asylum seekers living with HIV are lacking [18,19], likely owing to their minority status among asylum applicants and challenges in data collection stemming from HIV-related stigmatisation and confidentiality issues. Moreover, institutions that collect or register data on HIV diagnoses frequently categorise migrants by nationality, overlooking other essential characteristics such as the purpose of migration, the place of HIV acquisition or access to healthcare. Elucidating the demographic, clinical and virological profiles of asylum seekers is imperative to ensure optimal biomedical intervention and patient management and to facilitate the implementation of preventive measures. Thus, the objectives of this study were to describe the sociodemographic and clinical characteristics of Latin American asylum seekers living with HIV seeking international protection in Spain and to outline the barriers they face in seeking HIV treatment.

## Methods

### Study design and participants

This prospective cohort study was conducted between January 2022 and June 2023 at a single clinical site and registered at clinical trials (ClinicalTrials.gov NCT05998499). To be eligible for inclusion in the study, participants needed to meet the following criteria: they had to be asylum seekers from Latin America recently arrived in Spain, at least 18 years of age and diagnosed with HIV. Participants were recruited during the study period at the HIV outpatient clinic of Infanta Leonor University Hospital (HUIL) in Madrid, Spain and were monitored for a period of 6 months from enrolment (last participant data entry: June 2023).

Latin American migrants living with HIV who were seeking international protection in Spain (asylum seekers) were identified and referred to HUIL by various non-governmental organisations (NGOs) based in Madrid and working with migrants living with HIV. These NGOs established a crucial link, enabling patients to access the healthcare system through a direct route, bypassing the official or traditional referral pathways. Upon arrival at the hospital, asylum seekers were assigned a medical history number at the admissions service in the emergency department using their passports for identification. They were directly guided to the HIV clinic, where they were invited to participate in the study.

### Study procedures

#### Medical evaluation

In the HIV unit, assessments during the initial visit were conducted by infectious disease specialists and a nurse. The evaluation comprised a meticulous medical history, physical examination, and a series of routine tests (complete blood count, biochemistry panel, serology tests, urinalysis, swabs for sexually transmitted infections (STIs) and tuberculin test). The sociodemographic, clinical and laboratory data and details of HIV infection status collected from Latin American asylum seekers living with HIV included in the study are provided in Supplementary Table S2.

After the completion of these evaluations, patients were guided to the hospital pharmacy for dispensation of their ART. Furthermore, follow-up appointments were scheduled at 1 and 6 months to ensure continuous care and monitor the effectiveness of the treatment regimen (blood tests and evaluation of the clinical, social and administrative situation).

#### Social and psychological evaluation

An experienced social worker with a background in vulnerable populations was hired to perform a social assessment. Within 1 week following the HIV clinic visit, study participants were contacted and, once their consent had been obtained, a telephone or in-person interview was carried out to evaluate social aspects. Crucial dates related to the migratory journey, social determinants affecting the participants and barriers encountered in accessing the healthcare system, i.e. administrative access barriers, lack of information about care pathways, fear of deportation and stigma, were recorded during the interview. Additionally, they were asked to complete online questionnaires (as presented previously [20]) designed to evaluate various psychological (perceived stress, anxiety and depressive symptoms (HADS), stigma scale (HIV-related stigma score) and a scale that measures early childhood adverse events) and social aspects.

The social worker promoted engagement with the study and, after obtaining consent, collected multiple types of contact information (email, telephone number) to schedule reminders for appointments, thus helping to prevent loss to follow-up.

Six months after the initial HIV clinic visit, the social worker re-established contact with the study participants to reassess their social circumstances and their continued engagement with the HIV services.

### Data collection

Data for both the medical evaluation and social or psychological evaluation were collected using REDCap [21]. Clinical data were extracted from digitised medical records, while social data were sourced directly from interviews and entered into the database. The principal investigator checked the database for missing, invalid and inconsistent data. Questionnaires were self-completed by the participants, and the information was entered directly into REDCap. The database was stored securely on a dedicated server, with access restricted solely to the research team and designated health personnel involved in the project.

### Statistical analysis

We summarised baseline characteristics using descriptive statistics, with absolute and relative frequencies for categorical variables, and median and interquartile range (IQR) for continuous variables. In a univariate analysis, comparisons were made between participants’ characteristics and lack of ART and detectable HIV viral load at enrolment. The comparison was performed using the chi-square and the Mann–Whitney U tests. The methodology was based on a two-step analytical approach. First, a Poisson regression model was constructed to investigate the association between detectable HIV viral load and baseline characteristics, as well as barriers to healthcare. This was followed by an analysis to assess the absence of ART at the initial assessment. The multiple imputation by chained equations method was used, and a total of 50 imputations were carried out to account for missingness. This approach allowed us to obtain the incidence rate ratios. Variables were included if they were significantly associated with the dependent variable in the univariate analysis (p < 0.05). Additionally, age and gender were forced into the model to adjust for potential confounding effects. All analyses were performed using IBM SPSS Statistics for Windows, version 24.0 (IBM Corp., United States).

## Results

We evaluated 653 asylum seekers living with HIV who arrived in Spain between January 2022 and June 2023. Of these, 631 participated in the study and were included at the HIV clinic visit. The median age was 32 years (IQR: 28–37), 553 (88%) were cis men, 43 (7%) trans women and 35 (5%) cis women. In total, 581 (92%) were Hispanic and 48 (8%) were Black. The main countries of birth were Colombia (n = 189; 30%), Venezuela (n = 188; 30%) and Peru (n = 116; 18%). In total, 285 (45%) were university-educated, and 504 (80%) were employed in their country of origin before arriving in Europe.

Upon arrival, 309 participants (49%) lacked a social support network, i.e. family or acquaintances, 49 (8%) were homeless and slept in a migrant or homeless shelter and 464 (74%) experienced barriers when attempting to access the healthcare system (Table 1).

**Table 1:** Sociodemographic characteristics of HIV-infected Latin American asylum seekers evaluated in Madrid, Spain, January 2022−June 2023 (n = 631)

Characteristics	Number	Percentage
Age in years, median (IQR)	32 (28–37)	
Gender		
Cis women	35	5
Cis men	553	88
Trans women	43	7
Trans men	0	0
Race/ethnicity		
Black	48	8
Hispanic	581	92
Other	2	0
Country of birth		
Colombia	189	30
Venezuela	188	30
Peru	116	18
Cuba	21	3
Argentina	18	3
Honduras	17	3
Brazil	17	3
Other	65	10
Sexual orientation		
Homosexual	539	85
Heterosexual	61	10
Bisexual	20	3
Unknown	11	2
Educational level		
No formal education	2	0
Primary education	22	3
Secondary education	252	40
University education	285	45
Unknown	70	11
Employment status before arrival in Spain		
Active worker	504	80
Student	42	7
Unemployed	40	6
Retired or other	6	1
Unknown	39	6
Residence upon arrival		
Host home	557	88
Migrant shelter	14	2
Homeless shelter	35	6
Unknown	25	4
Social support network		
Family	113	18
Friends	177	28
None	309	49
Unknown	32	5
Right to healthcare at first evaluation		
Yes	192	30
No	344	55
Unknown	95	15
Barriers to access to the healthcare system		
Yes	464	74
No	66	10
Unknown	101	16

IQR: interquartile range.

Categories labelled as ‘Unknown’ represent data that were not available at the time of assessment.

Of all the participants, 460 (73%) were sexually active and 155 of these (34%) engaged in unprotected anal sex outside their stable relationship. In the previous year, 299 (47%) had been diagnosed with a sexually transmitted infection (STI), with syphilis being the most common, accounting for 256 (86%) of the 299 participants. A total of 81 (13%) participants had consumed drugs in the previous year, 47 (7%) used drugs in the context of chemsex and 17 (3%) administered drugs intravenously. Supplementary Table S1 provides details on other risk behaviours reported by the participants.

### HIV infection status

All study participants in the cohort had been diagnosed with HIV infection (median duration of infection 5 years (IQR: 3–8) and 97% (n = 610) had acquired the virus through sexual relations (Table 2). Of all 631 participants, the median nadir CD4^+^ T lymphocyte count was 297 cells/mm (IQR: 190–450) and 13% (n = 81) had had an opportunistic infection, mainly tuberculosis (38/81; 47%).

**Table 2:** Factors related to HIV infection in Latin American asylum seekers evaluated in Madrid, Spain, January 2022−June 2023 (n = 631)

Factors	Number	Percentage
Characteristics		
Duration of HIV infection in years, median (IQR)	5 (3–8)	
**Transmission**		
Sexual	610	97
Sharing needles or syringes	1	0
Mother-to-child	3	0
Unknown	17	3
**AIDS-related events**		
Nadir CD4+ T lymphocytes, median cells/mm (IQR)	297 (190–450)	
Previous opportunistic infection	81	13
Tuberculosis	38	6
Toxoplasmosis	12	2
*Pneumocystis jirovecii *pneumonia	10	2
HIV wasting syndrome	10	2
Recurrent bacterial pneumonia	8	1
Candidiasis of bronchi, trachea, oesophagus or lungs	9	1
Kaposi's sarcoma	8	1
Histoplasmosis	7	1
Cryptococcosis	5	1
Herpes simplex	4	1
Lymphoma	1	0
Cytomegalovirus disease	2	0
Chronic intestinal cryptosporidiosis	1	0
**ART**		
ART-naïve	90	14
Had taken ART previously	541	86
**Last ART based on:**		
Non-nucleoside reverse transcriptase inhibitors	280	52
Integrase inhibitors	183	34
Protease inhibitors	30	6
Unknown	48	9
Taking ART upon arrival in Spain	487	77
Initial HIV clinic evaluation		
Continued taking ART at the first visit	386	61
Weeks without ART, median (IQR)	4 (2–10)	
CD4+ T lymphocytes, median cells/mm (IQR)	500 (349–689)	
Percent CD4+	28 (20–34)	
Detectable viral load (> 30 copies/mL)	209	33
Log10 of viral load (IQR)	4.1 (2.8–4.9)	
Viral load > 1,000 copies/mL	154	24
Resistance test performed	150	24
Valid resistance test	142	22
Mutations to reverse transcriptase inhibitors	53	37
Mutations to protease inhibitors	7	15

ART: antiretroviral therapy; IQR: interquartile range.

### Antiretroviral therapy

A total of 541 study participants (86%) had previously been taking ART, while 90 (14%) were ART-naïve. Upon arrival in Spain, 77% (n = 487) were taking ART, although this decreased to 61% (n = 386) because of barriers to treatment access by the time they were evaluated at the HIV clinic (Figure). Information on study participants including country of birth and whether they continued taking ART at the initial assessment is provided in Supplementary Table S3.

**Figure:**
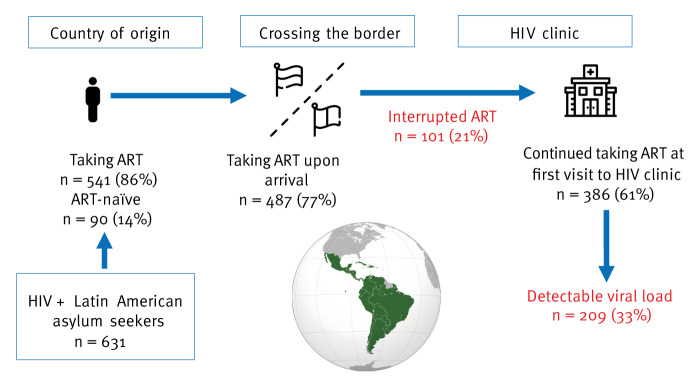
Prevalence of participants on antiretroviral therapy during each phase of the migration journey to Spain, January 2022−June 2023 (n = 631)

ART: antiretroviral therapy.

The median time from arrival in Spain to HIV clinic evaluation was 8 weeks (IQR: 4–17). During the evaluation at the HIV clinic, 33% (n = 209) of the participants in the cohort had a detectable viral load (HIV-1 RNA > 50 copies/mL). For study participants who discontinued ART after arrival, the median duration of treatment interruption was 4 weeks (IQR: 2–10). During the initial clinical evaluation, the median CD4^+^ T lymphocyte count of all participants was 500 cells/mm (IQR: 349–689) and the median plasma viral load was 4.1 log_10_ (IQR: 2.8–4.9).

Among the 541 study participants who were taking or had taken ART, 52% (n = 280) had taken non-nucleoside reverse transcriptase inhibitor (NNRTI)–based regimens, 34% (n = 183) integrase inhibitors and 6% (n = 30) protease inhibitors. Unknown regimens accounted for 9% (n = 48). Common regimens included efavirenz/tenofovir/lamivudine (EFV/TDF/3TC) (151/534; 28%) and dolutegravir/l/tenofovir/lamivudine (DTG/TDF/3TC) (89/534; 17%). Resistance testing was conducted in those with a viral load > 1,000 copies/mL (n = 154; 74% of those with a detectable viral load). In 12 study participants (8%), it was not possible to carry out the resistance testing owing to successive inhibitions of the amplification reaction. Among the 142 participants with a valid resistance test, 53 (37%) had mutations to NNRTIs and 7 (5%) had mutations to protease inhibitors (Table 2).

### Factors associated with lack of viral control

The initial evaluations showed detectable viral load to be associated with being transgender (11% (n = 23) vs 5% (n = 20); p = 0.003), lacking university education (56% (n = 103) vs 46% (n = 173); p = 0.031), homelessness (12% (n = 24) vs 6% (n = 25); p = 0.012), drug use (21% (n = 42) vs 9% (n = 39); p < 0.001), and not taking ART (79% (n = 166) vs 19% (n = 79); p < 0.001). The multivariate analysis, provided in Supplementary Figure S1, highlighted not taking ART as a primary factor for detectable viral load. Conversely, the only variable associated with not continuing taking ART at the initial visit was drug use in the previous year (74% vs 58%, p = 0.015). This association remained significant after adjusting for other variables in the multivariate analysis. Those who had used drugs were 1.6 times more likely to attend the consultation without taking ART (95% confidence interval (CI): 1.16–2.12; p = 0.003).

### Retention in care after 6 months

Six months after an initial visit, a social worker attempted to contact all participants, and was able to reach 520 (82%) study participants: 459 (88%) by phone and 61 (12%) in person; 111 (18%) were not able to be contacted. Follow-up data were available (through contact with the participant or by reviewing the medical record) for 603 study participants (96%). However, of these, 130 (21%) were lost to follow-up care. The main reasons were unknown in 70 (54%) participants, relocation in 59 (45%) and death in one participant (1%). At 6 months, 515 (99%) participants were still taking ART, and 509 (98%) maintained an undetectable HIV viral load. Regarding social health determinants, 277 (54%) of 516 participants secured healthcare entitlements within 6 months, and 203 (40%) of 513 found employment.

## Discussion

This study presents data on a prospective cohort of Latin American asylum seekers living with HIV in Madrid, a leading destination in Europe for migrants from South and Central America. Migrants living with HIV who seek asylum and international protection in Spain consist primarily of young, educated gay men. They often flee their home countries because of severe human rights violations, which are frequently related to their HIV status or sexual orientation [22]. This background, coupled with the challenge of migration, heightens their vulnerability. Among those taking ART upon arrival in Spain, 21% had interrupted their treatment before being evaluated at the HIV clinic. Despite their status as asylum seekers and their use of direct pathways for treatment through NGOs, a third of those evaluated at the HIV clinic had a detectable HIV viral load. Much of the research on the HIV epidemic in European centres has been conducted on sub-Saharan and Eurasian migrants [23,24]. There is a lack of data on people from Latin America and it is important to recognise the distinct characteristics of individuals coming from this continent. Our study is to the best of our knowledge, the first to specifically focus on those from Latin America and provides important insights into asylum seekers living with HIV coming to Europe.

In 2022, a particular interpretation of the law by the regional government resulted in asylum seekers in Madrid facing restricted access to the healthcare system, where they were classified as tourists. During this period, only two public hospitals, Hospital Ramón y Cajal and HUIL, granted healthcare access to these individuals because of their internal policies. Consequently, many NGOs referred HIV-infected asylum seekers to our institution. Following evaluation by the NGO, the process of establishing contact and facilitating referral to a hospital was executed with immediacy (< 1 week). In our study, more than one in five asylum seekers faced interruptions in their medical treatments upon arrival in Spain. The majority of participants interrupted their medication regimen during the interval between their arrival in the country and their assessment by an NGO.

While barriers such as socioeconomic constraints, HIV-related stigmatisation, fear of deportation and language or cultural differences have been identified [25,26], the most substantial obstacle highlighted in our study was the administrative challenge. It is important to emphasise that all asylum seekers included in this study could not access healthcare services through traditional pathways and instead relied on NGOs for their healthcare needs. Even though healthcare access is a basic right in Spain [27,28], administrative issues such as complex registration processes or restrictive policies can prevent migrants and asylum seekers, especially new arrivals, from using these services. Despite Spain's efforts towards universal healthcare, exemplified by Law 7/2018 [29,30], regional disparities and variations in application of the law pose challenges, especially for recently arrived migrants [13]. These disparities highlight the possible public health implications of administrative obstacles, given the elevated risk of HIV transmission among such a vulnerable group.

In our study, a concerning 33% of participants had detectable HIV viral load upon consultation. Given the WHO targets and the inherent vulnerability of this group, such a rate is concerning and indicates a need for enhanced intervention. Our research indicates the impact of barriers on HIV viral load detection rates. This has implications, both for individual health and wider public health considerations in Madrid and Spain. The active involvement of institutions and regional health services is important to overcome administrative obstacles.

Research from the advancing Migrant Access to health services in Europe (aMASE) indicates that up to 71% of HIV-positive migrants in Europe contracted HIV after migrating [31], thus underscoring the pressing need for tailored sexual health education and preventive interventions in this population [23]. Our data highlighted frequent involvement in high-risk behaviours among HIV-positive asylum seekers from Latin America, culminating in a marked incidence of STIs and other unsafe sexual practices. In our study, 47% of participants reported an STI, 13% used drugs, 7% in chemsex contexts, and 25% had unprotected anal intercourse over the past year. Comparatively, the U-SEX-2 GESIDA 9416 multicentre study in Madrid found that 33% of HIV-positive MSM were diagnosed with an STI, with 25% practicing chemsex [32]. These findings suggest a high STI prevalence among both asylum seekers and local patients, although recreational drug use and unprotected anal sex were less prevalent among the asylum seekers from Latin America.

We found that the medical care of asylum seekers is often complicated by the absence of a detailed medical history and unfamiliarity with prior treatments upon arrival. Most asylum seekers predominantly relied on low genetic barrier NNRTI-based regimens. Asylum seekers often had to interrupt their HIV treatment, due to lack of medication stock in their home countries, challenges during the migratory journey, or limited access in Madrid. This interruption places them at a higher risk of virological failure and accumulation of resistance mutations. Our data point to a considerable number of asylum seekers in our study having resistance mutations specific to NNRTIs. This high prevalence (37% for NNRTIs) is critical when considering the initiation or resumption of HIV treatment, as has been documented in other studies of migrants in various European countries [33,34].

In our study, following the integration of asylum seekers into HIV clinic care, the observed 6-month follow-up attrition rate stood at 21%, a figure within anticipated ranges for this demographic cohort. The results from the 6-month follow-up are promising, because, as we have seen in our study, once access to the healthcare system was secured, antiretroviral therapy and retention in care were also ensured. Nonetheless, this rate differs from the loss to follow-up data of the Spanish population with HIV between 2004 and 2020, which found that 35.9% had at least one medical care interruption [35]. In this study, being born in another country was identified as a factor associated with an increased likelihood of loss to follow-up. Once migrants access treatment, they often achieve an undetectable viral load. Moreover, many managed to acquire resident status in Spain, finding stability and employment. This outcome highlights the resilience of the migrant population living with HIV and the potential benefits when barriers to care and integration are effectively addressed.

Our study examines the characteristics of asylum seekers from Latin America arriving in Madrid and analyses the barriers they encounter in accessing the healthcare system. It emphasises the vital role of NGOs in bridging healthcare access gaps and suggests a need for policy interventions and collaborative efforts between healthcare systems, NGOs, and migrant communities. This approach is crucial for healthcare inclusivity and reducing HIV viral load detectability.

Our study was subject to a series of limitations. Firstly, the fact that participants were recruited primarily from an HIV clinic via NGOs could introduce selection bias. However, in 2022, Madrid was the primary destination for most Latin American asylum seekers arriving in Spain, and possibly even in Europe. Our centre was the only one employing this referral pathway in Madrid, thus making the findings generalisable to the broader Latin American population with HIV in the area. Secondly, our results could be subject to recall bias, as many participants lacked medical records, were unfamiliar with their previous treatments, or could not remember past medical information. To mitigate this, we conducted a thorough baseline assessment for each participant. The shared language facilitated in-depth conversations, and in some instances, we even reached out to medical centres in the countries of origin to gather data. The transient nature of this population meant that there were frequent changes in their exposure and characteristics. While loss to follow-up and difficulties in contacting some participants were inherent limitations of a real-world, dynamic cohort, the size of our cohort helps to offset these biases.

## Conclusions

Our study indicates that Latin American asylum seekers living with HIV encounter difficulties accessing healthcare upon their arrival in Madrid, Spain. Even with direct referral pathways, one-third presented with detectable HIV viral loads during clinical evaluations. These findings emphasise the urgent need to enhance access and provide tailored care for this population group, considering the broader public health implications.

## Supplementary Data

321000 Supplement
