# Supplementary material for: HIV-infected Latin American asylum seekers in Madrid, Spain, 2022: A prospective cohort study from a major gateway in Europe
Source: Euro Surveill. 2024 Jul 18;29(29):2300692. doi: 10.2807/1560-7917.ES.2024.29.29.2300692 (PMC11258946; doi:10.2807/1560-7917.ES.2024.29.29.2300692)
Supplement: Supplement [file 23-00692_RYAN_Supplement.pdf]

Supplementary material

This supplementary material is hosted by Eurosurveillance as supporting information alongside the article “HIV infected Latin American asylum seekers in Madrid, Spain, 2022: A prospective cohort study from a major gateway in Europe” on behalf of the authors who remain responsible for the accuracy and appropriateness of the content.

The same standards for ethics, copyright, attributions, and permissions as for the article apply.

Eurosurveillance is not responsible for the maintenance of any links or email addresses provided therein.”

Table S1: Drug use, risky sexual behaviours, and STIs in study participants.

Table S2: Sociodemographic, clinical and laboratory data and details of HIV infection status collected from Latin American asylum seekers living with HIV included in the study.

Table S3: Study participants by country of birth and whether they were on art at the initial assessment.

Figure S1: Factors associated with having a detectable viral load and not taking ART at inclusion.

Table S1: Drug use, risky sexual behaviours, and STIs of study participants.

**Characteristics in the last year**	**N=631**
Used drugs, n (%)	81 (13)
Cocaine	36 (6)
Cathinones	35 (6)
Methamphetamine	23 (4)
Ketamine	13 (2)
Gamma Hydroxybutyrate	29 (5)
Ectasis	12 (2)
Heroine	2 (0)
Other	26 (4)
Injected drugs, n (%)	17 (3)
Context of drugs use, n (%)	
Non-sexual	24 (4)
Sexual	35 (5)
In both context	12 (2)
Stable partner, n (%)	214 (33)
HIV infected partner, n (%)	95 (44)
Sexually active, n (%)	460 (73)
Sexual partners/month*, median (IQR)	1 (1-3)
Engaged in UAI*	155 (25)
Diagnosed with STI, n (%)	299 (47)
Syphilis	256 (41)
Gonorrhoea or chlamydia.	94 (15)
Hepatitis C	9 (1)
Lymphogranuloma venereum	7 (1)

Abbreviations: STI, sexually transmitted infection; GHB, *, outside of a stable relationship; IQR, interquartile range; UAI, unprotected anal intercourse.

Table S2: Sociodemographic, clinical and laboratory data and details of HIV infection status collected from Latin American asylum seekers living with HIV included in the study.

Date of Inclusion
HIV Infection
Asylum Seeker
Irregular Situation
Student
Gender
Date of Birth
Age in Years
Race
Country of Birth
Your Educational Level
Employment Situation Before Traveling to Spain
Sexual Orientation
Stable Partner
Does Your Partner Have HIV?
Date of Arrival in Spain
Residence Upon Arrival in Spain
Lives in a Household With
Social Support Network
Right to Healthcare Assistance
Do You Have Barriers to Accessing the Healthcare System?
Type of Barriers
Date of First Appointment in Processing Unit
Date of Obtaining Permission (DASSE, DASPI, etc.)
Date of First Evaluation in HIV Consultation
Have You Used Drugs in the Last Year?
Cocaine
Cathinones (Mephedrone, etc.)
Methamphetamines (Tina, etc.)
Ketamine
GHB
Ecstasy
Heroin
Other Drugs
Have You Injected Any of These Drugs?
In What Context Have You Used These Drugs?
Sexually Active (Last Year)
Number of Sexual Partners per Month
Have You Practiced Unprotected Anal Sex in the Last Year? (Outside of a Stable Relationship)
Have You Used Drugs During Sexual Intercourse? (Chemsex)

In the Last Year, Have You Been Diagnosed With a Sexually Transmitted Infection? (e.g., Syphilis, Chlamydia, Gonorrhea, Hepatitis C, Genital Herpes, etc.)
Syphilis
Urethritis Due to Gonorrhea or Chlamydia
Acute Hepatitis C
Lymphogranuloma Venereum
Year of HIV Diagnosis
HIV Transmission
Previous Opportunistic Diseases
Opportunistic Diseases
Nadir CD4
Have You Taken Antiretroviral Treatment Before Being Assessed in the First Consultation?
When You Arrive in Spain, Do You Take ART?
When You Attend the First Visit, Do You Take ART?
ART That the Patient Was Taking Upon Arrival
What Other ART Was the Patient Taking Upon Arrival
Year of Starting ART
Weeks Without Treatment
HIV Viral Load in Baseline Analysis
CD4+ Lymphocytes
ART Prescribed in Consultation
What Other ART Are You Taking
Date of the Analysis
Undetectable HIV Viral Load
HIV Viral Load
Mutations in Reverse Transcriptase Inhibitors
Mutations in Protease Inhibitors
Haemoglobin
Haematocrit
MCV (Mean Corpuscular Volume)
Lymphocytes
Eosinophils
CD4 T Lymphocytes
Percentage of CD4 T Lymphocytes
CD8 T Lymphocytes
Percentage of CD8 T Lymphocytes
CD4/CD8 Ratio
Platelets
HIV Serology
HBsAg (Hepatitis B Surface Antigen)
Anti-HBc (Hepatitis B Core Antibody)
Anti-HBs >10 (Hepatitis B Surface Antibody)
HCV Serology (Hepatitis C Virus)
Varicella Zoster Virus IgG Antibody

Measles IgG Antibody
Rubella IgG Antibody
Mumps IgG Antibody
Toxoplasmosis IgG Antibody
Toxoplasmosis IgM Antibody
Leishmania IgG Antibody
Treponemal Test
RPR (Rapid Plasma Reagin)
RPR Titer
Glucose
Creatinine
Uric Acid
Urea
Sodium
Potassium
Magnesium
Phosphorus
Total Proteins
Calcium
Albumin
Total Bilirubin
LDH (Lactate Dehydrogenase)
ALT (Alanine Aminotransferase)
AST (Aspartate Aminotransferase)
C-reactive Protein
LDL Cholesterol (Calculated)
Triglycerides
Iron
Transferrin
Ferritin
Folic Acid
Vitamin B12
Beta-2 Microglobulin
Alpha-fetoprotein
Pittsburgh Sleep Quality Index
HADS (Hospital Anxiety and Depression Scale)
Loss to Follow-up
Reason for Loss to Follow-up
Date of Visit
Type of Visit
Continues with ART
Presents Undetectable HIV Viral Load in Plasma
Have You Achieved the Right to Healthcare Assistance?
Have You Found Employment or Are You Currently Working?
Have You Regularized Your Situation?
Reviewed by Social worker

Table S3: Study Participants by Country of Birth and Whether They Were on ART at the Initial Assessment

	Total	On ART		Without ART	
**Country**	**N**	**N**	**%**	**N**	**%**
Colombia	189	106	56.10%	83	43.90%
Venezuela	188	124	66.00%	64	34.00%
Peru	116	79	68.10%	37	31.90%
Cuba	21	10	47.60%	**11**	**52.40%**
Argentina	18	10	55.60%	8	44.40%
Brasil	17	10	58.80%	7	41.20%
Honduras	17	6	35.30%	**11**	**64.70%**
El Salvador	12	8	66.70%	4	33.30%
Mexico	11	8	72.70%	3	27.30%
Paraguay	8	4	50.00%	**4**	**50.00%**

This table shows the top 10 countries contributing the most participants (94% of the total sample).

Figure S1: Factors associated with having a detectable viral load and not taking ART at the inclusion of the participants.

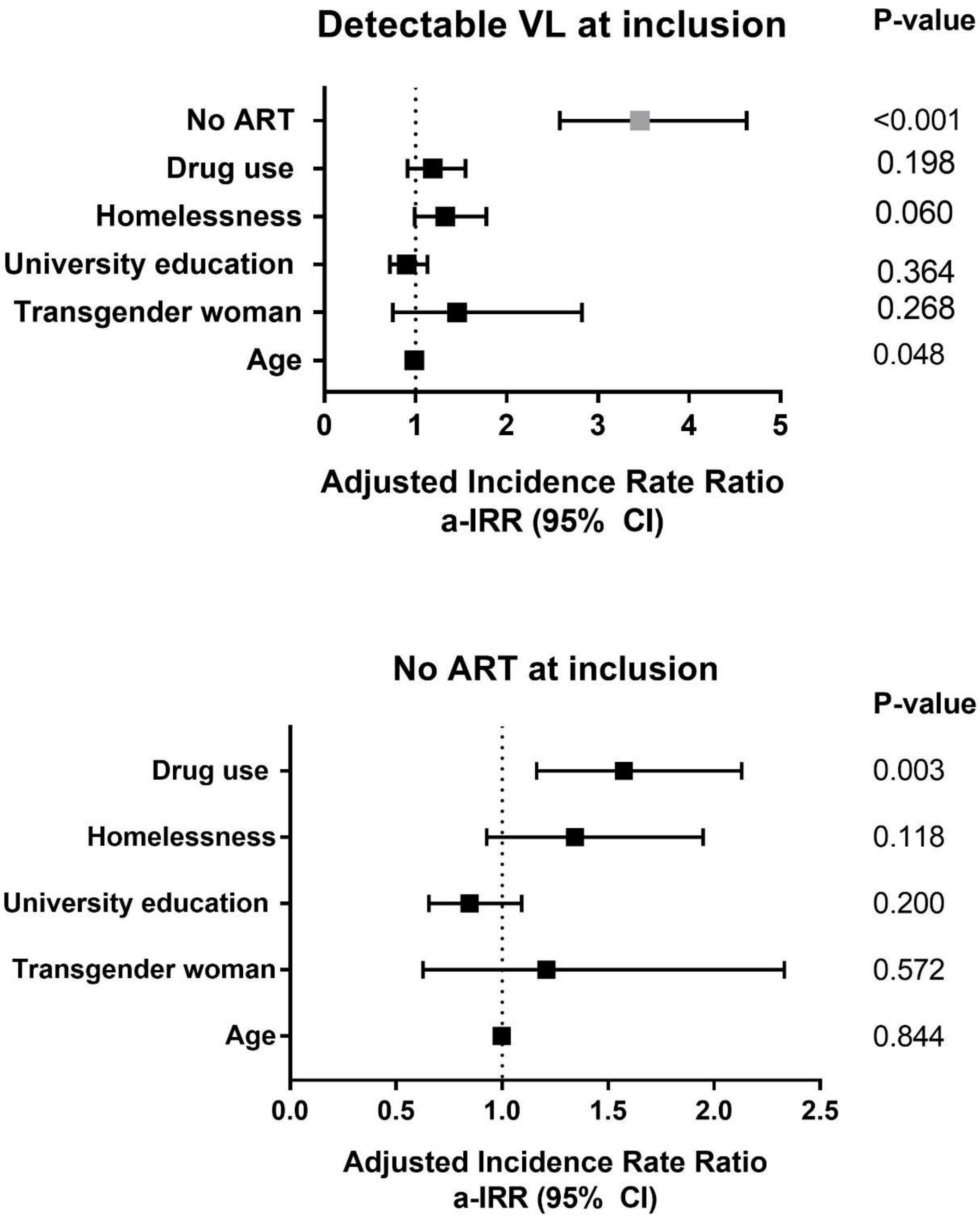

Abbreviations: VL; viral load; a-IRR, adjusted incidence rate ratio; CI, confidence interval; ART, antiretroviral therapy.
